# An efficacy and safety evaluation of montelukast + fluticasone propionate vs. fluticasone propionate in the treatment of cough variant asthma in children: a meta-analysis

**DOI:** 10.1186/s12890-023-02721-z

**Published:** 2023-12-05

**Authors:** Zhengbo Wei, Sheng Li

**Affiliations:** Department of Pediatrics, The Affiliated Yancheng Maternity&Child Health Hospital of Yangzhou University Medical School, No. 31, Century Avenue East Road, Economic Development Area, Yancheng, Jiang Su Province 224002 China

**Keywords:** Cough variant asthma, Fluticasone propionate, Meta-analysis, Montelukast

## Abstract

**Purpose:**

This study aimed to evaluate the efficacy and safety of montelukast (Mon) + fluticasone propionate (Flu) versus Flu in the treatment of cough variant asthma (CVA) in children.

**Methods:**

Eligible documents were selected from various databases. Weighted mean difference (WMD) and 95% confidence interval (CI) were used to evaluate continuous variables, and categorical variables were evaluated using risk ratio (RR) and 95% CI. Heterogeneity analysis was performed using Cochran’s Q test and *I*^*2*^ statistics, followed by sensitivity analysis and publication bias evaluation.

**Results:**

Nine studies were included, and Flu + Mon was found to significantly improve the total effective rate and reduce cough recurrence compared to Flu. The cough remission and disappearance times in the Mon + Flu group were significantly lower than those in the Flu group. FEV1% recovery in the Mon + Flu group was significantly better than that in the Flu group.

**Conclusion:**

Mon + Flu is effective and safe for the treatment of CVA in children.

**Supplementary Information:**

The online version contains supplementary material available at 10.1186/s12890-023-02721-z.

## Introduction

Cough variant asthma (CVA), a phenotype of asthma that exhibits predominantly or solely airway hyperresponsiveness and cough but without wheezing or dyspnea [1, 2], is the most common cause of chronic cough [3]. Patients with CVA tend to experience hidden onsets, long courses, and repeated illnesses that directly affect their study, life, work, and sleep, and heavily affect their economic and mental well-being [4]. Induction therapy consisting of beta2-agonists, leukotriene receptor antagonists, and inhaled corticosteroids is considered useful for CVA [5]. However, resistance to therapy leads to a false-negative result, causing 30% of patients with CVA to develop typical bronchial asthma within a few years [6]. Therefore, it is of great significance to explore therapeutic methods with good curative effects and safety for the clinical intervention of CVA.

Inhaled glucocorticoids, such as fluticasone propionate (Flu) and oral leukotriene receptor antagonists, including montelukast (Mon), are commonly used for the clinical treatment of CVA [7]. Airway hyper-responsiveness due to chronic inflammation is believed to be the underlying mechanism of the disease. Leukotriene receptor antagonists are anti-asthma medications with anti-inflammatory and bronchodilatory properties [8]. A previous study showed that the leukotriene receptor antagonist Mon alone was effective for the treatment of CVA [9]. Furthermore, Takemura et al. indicated that the antitussive effect of Mon alone in CVA could be attributed to the attenuation of eosinophilic inflammation [10]. Moreover, a previous study showed that after two months of treatment, the Flu group had a significantly lower cough symptom score, which is a sample two-part questionnaire relating to cough symptoms [11] and significantly higher forced expiratory volume in 1 s (FEV1%) and percentage of predicted peak expiratory flow (PEF%) than the control group, indicating the effect of Flu treatment on CVA [12]. Ostrom et al. reported that Flu was significantly more effective than Mon in improving pulmonary function, asthma symptoms, and rescue albuterol use [13]. A recent randomized controlled trial (RCT)study explored the differences in the efficacy and safety of Mon combined with Flu or Flu alone in the treatment of CVA in children; however, the conclusions were inconsistent [12, 14, 15]. Therefore, it is necessary to comprehensively evaluate the efficacy and safety of Mon + Flu vs. Flu in the treatment of CVA in children based on a meta-analysis.

A meta-analysis is a statistical procedure that integrates the results of several independent studies that are considered combinable [16]. Although a meta-analysis has explored the relationship between Mon and Flu in CVA [17], some shortcomings cannot be ignored in previous studies, such as publication bias [18]. To obtain more comprehensive and objective results, current study has revealed the efficacy and safety of Mon + Flu vs. Flu in the treatment of CVA based on an updated meta-analysis. This study may provide novel insights into the clinical treatment of CVA.

## Materials and methods

This meta-analysis was performed according to the Preferred Reporting Items for Systematic Reviews and Meta-Analyses (PRISMA) statement guidelines (Table S1).

### Data sources

Relevant studies were searched from electronic databases, including PubMed, Embase, WanFang, China National Knowledge Infrastructure (CNKI), and China Science and Technology Journal Database (CQVIP). The main searching keywords included: “Montelukast,” “fluticasone,” “fluticasone propionate,” “cough variant asthma,” and “CVA”. Keywords of the same category were combined with “or,” while keywords of different categories are combined with “and.” We combined subject words with free words to search and adjust the search mode according to the database characteristics. Additionally, the paper versions of the literature results were manually investigated, and relevant reviews and references were screened. The retrieval time for the present study was updated on July 3, 2022. No language restrictions were imposed on the meta-analysis.

### Inclusion and exclusion criteria

The inclusion criteria for selected studies were as follows: i) patients with variant asthma under 18 years old; ii) study focused on the differences between Mon + Flu and Flu; iii) studies that were designed as RCTs; and iv) studies that included one or more of the following outcomes: treatment efficiency, cough recurrence rate, cough symptom score, cough remission time, cough disappearance time, FEV%, PEF%, and adverse reactions. In addition, the exclusion criteria were as follows: i) non-treatise literature, such as reviews, letters, and comments; ii) randomized controlled trials, such as cohort, case-control, and cross-sectional studies; and iii) duplicate publications or those that used the same data for multiple articles (only the one with the most complete data was retained).

### Data extraction and quality assessment

Two investigators independently extracted available articles according to the selection criteria. Next, the following information was collected from each eligible study independently: first author, year of publication, recruitment time, treatment cycle and follow-up time, treatment plan and dose, basic information of subjects (sample size, age, sex, course of disease), and outcome. Both investigators reached a consensus on all items via discussion and re-examination.

The quality assessment of the included studies was based on the Cochrane guidelines [19], an official document that describes in detail the process of preparing and maintaining Cochrane systematic reviews on the effects of healthcare interventions. If disputes arose during the data extraction and quality assessment, a panel discussion was held, and a third investigator was consulted to obtain consistent results.

### Statistical analysis

Stata software (version 12.0) and RevMan (version 5.3) were used for statistical analysis. WMD (weighted mean difference) and 95% confidence interval (CI) were used to evaluate the continuous variables. The risk ratio (RR) and 95% CI were used to evaluate categorical variables. Heterogeneity analysis was performed using Cochran’s Q test and *I*^*2*^ statistics [20]. A random-effects model was used if heterogeneity was observed (P < 0.05, *I*^*2*^ > 50%); otherwise, a fixed-effects model was used (P ≥ 0.05, *I*^*2*^ ≤ 50%). Meta-regression was used to evaluate the effects of sample size and treatment cycle on heterogeneity. Moreover, a sensitivity analysis was performed to evaluate whether the combined outcomes were affected by the removal of a single study. Finally, publication bias was evaluated using a funnel plot and Egger’s test [21].

## Results

### Included studies

**Fig. 1:**
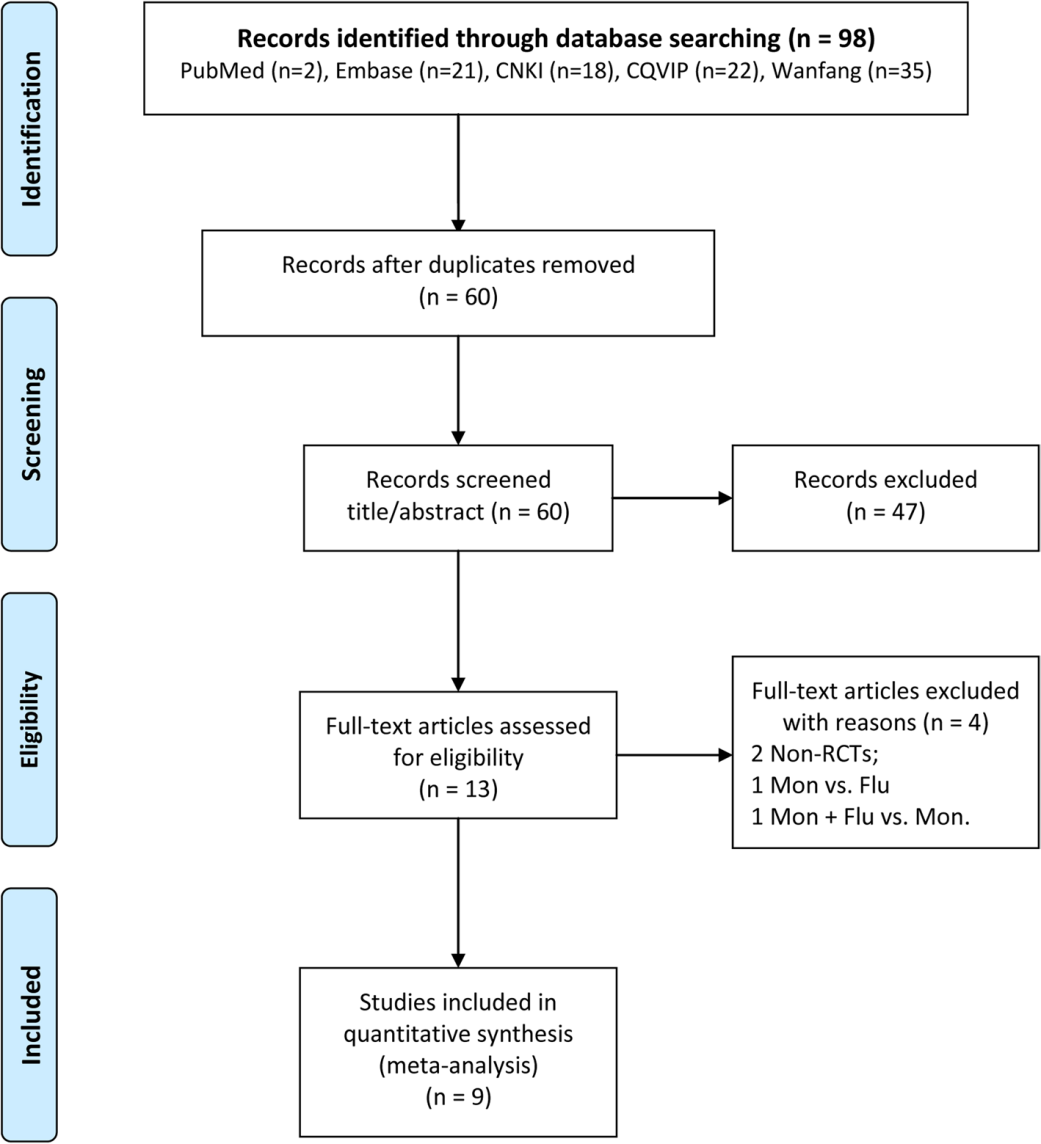
Flow diagram of the screening process for eligible articles. 98 studies from all enrolled databases were initially retrieved. After eliminating 38 duplicates, 60 articles left. Then 47 articles were excluded after reading titles and abstracts, 4 were excluded after reading the full text. lefting nine studies included into meta-analysis

As shown in Fig. 1, the present meta-analysis initially retrieved 98 studies from all enrolled databases. After eliminating 38 duplicates, 60 studies were retained. After reading titles and abstracts, 47 articles were excluded because they did not meet the inclusion criteria. From the remaining 13 studies, four were filtered out after reading the full text. However, the manual search failed to identify studies that could be included in the current analysis. Finally, nine studies [14, 15, 22–28] with sufficient data were enrolled in the present meta-analysis.

### Characteristics of the included studies

A total of 926 patients from the nine studies were enrolled, including 464 and 378 patients in the Mon + Flu and Flu groups, respectively (Table 1). There was no significant difference in sex composition (500 boys and 426 girls), age, and course of disease between the two groups. The study area was mainly located in China. The diagnostic criteria of the subjects were the same, and the children did not receive glucocorticoids, leukotriene antagonists, antihistamines, or other drugs within one month before the start of the study. With the exception of Wu et al. [28] and Zeng et al. [23], the dosages used in other studies were the same. The median treatment period of each study was 12 (8-24) weeks, and the follow-up time ranged from 3-6 months.

**Table 1:** Characteristics of 9 included studies in this meta-analysis

Study	Duration	N	Treatment cycle, weeks	Follow-up, months	Group	n, M/F	Age, years	Course of disease, months	Dose
Chen, JC 2016 [24]	2013.02-2015.01	160	12	6	Mon+Flu	80, 43/37	6.3±0.7	4.7±0.5	Flu group + 4-5 mg/day
					Flu	80, 41/39	6.5±0.9	4.8±0.8	2 times/day, 125 μg/time
Dong, JJ 2020 [25]	2016.06-2018.06	70	12	3	Mon+Flu	35, 20/15	6.8±1.8	6.0±2.2	Flu group + 4-5 mg/day
					Flu	35, 20/15	6.6±1.2	5.4±1.5	2 times/day, 125 μg/time
Hu, HJ 2021 [26]	2017.01-2019.12	100	12	NR	Mon+Flu	50, 27/23	7.1±2.2	2.08±0.60	Flu group + 4 mg/day
					Flu	50, 27/23	7.5±2.4	2.18±0.62	2 times/day, 125 μg/time
Lai, M 2021 [14]	2017.07-2020.01	64	8	NR	Mon+Flu	32, 19/13	8.97±2.54	3.68±0.74	Flu group + 5 mg/day
					Flu	32, 16/16	9.10±2.17	3.66±0.81	2 times/day, 125 μg/time
Li, RX 2018 [27]	2016.01-2016.12	97	12	6	Mon+Flu	49, 29/20	8.92±1.07	10.32±4.56	Flu group + 4-5 mg/day
					Flu	48, 30/18	9.11±0.87	9.79±5.02	2 times/day, 125 μg/time
Wu, JH 2011 [28]	2004.07-2008.01	85	12	6	Mon+Flu	43, 23/20	6.2±3.6	2.47±1.26	Flu group + 4-5 mg/day
					Flu	42, 23/19	5.8±4.3	2.66±1.18	1-2 times/day, 125 μg/time
Xu, QR 2018 [15]	2013.06-2016.01	180	8	NR	Mon+Flu	90, 47/43	10.38±2.25	NR	Flu group + 5 mg/day
					Flu	90, 42/48	12.00±1.74	NR	2 times/day, 125 μg/time
Zeng, JZ 2016 [23]	2014.03-2016.03	90	24	NR	Mon+Flu	45, 27/18	5.9±0.3	12.4±3.3	Flu group + 10 mg/day
					Flu	45, 26/19	5.6±0.5	13.2±3.7	2 times/day, 250 μg/time
Zhu, XH 2019 [22]	2015.06-2018.01	80	12	3	Mon+Flu	40, 19/21	5.9±0.9	5.4±0.5	Flu group + 4-5 mg/day
					Flu	40, 21/19	6.0±0.9	5.3±0.7	2 times/day, 125 μg/time

*Flu *Fluticasone propionate, *Mon *Montelukast sodium, *F *Female, *M *Male, *NR *Not reported

**Fig. 2:**
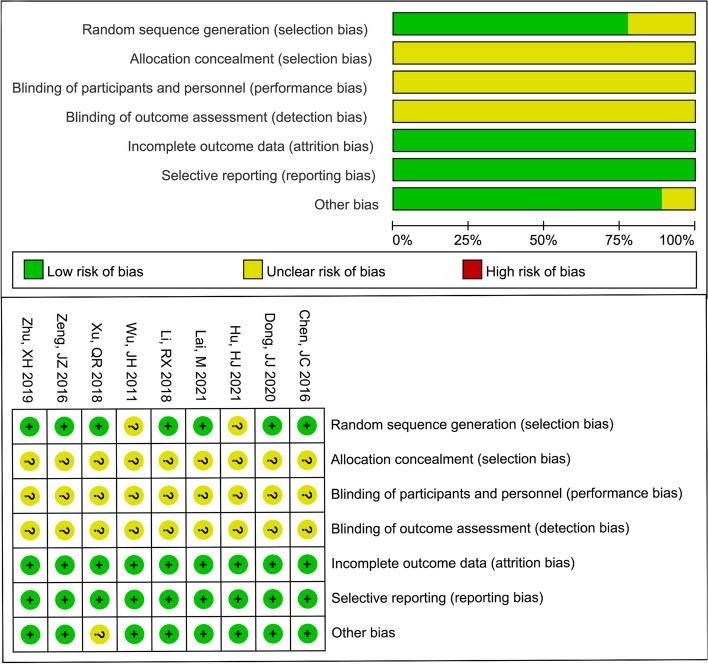
The results of the quality evaluation for the current analysis. Green indicates low risk of bias; yellow indicates unclear risk of bias and red indicates high risk of bias. Overall, the quality of the methodology used in this study was medium

The results of the literature quality evaluation showed that some of the included studies had moderate risk bias in the evaluation of selection bias, performance bias, and detection bias. Overall, the quality of the methodology used in this study was medium. The detailed characteristics and quality assessment of the eligible studies are shown in Fig. 2.

### Results of meta-analysis

**Fig. 3:**
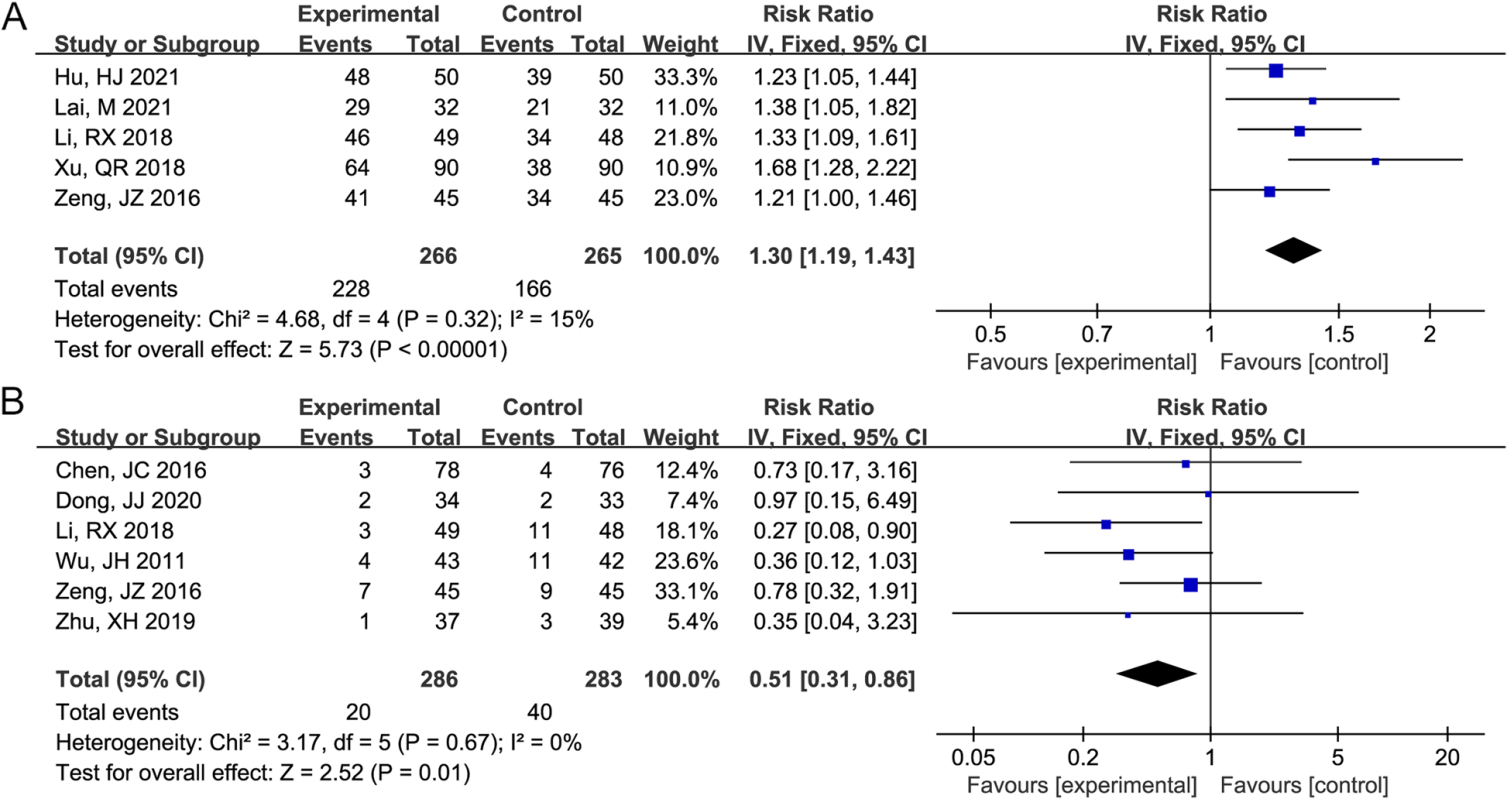
The result of the forest plot analysis for the total effective rate and cough recurrence rate between the montelukast (Mon) + fluticasone propionate (Flu) group and the Flu group. **A** The result of the forest plot analysis for total effective rate between the Mon + Flu group and the Flu group. A fixed-effect model was used since the I^2^=15%. The pooled results showed that Flu + Mon significantly improved the total efficacy rate. **B **The result of the forest plot analysis for cough recurrence rate between the Mon + Flu group and the Flu group. A fixed-effect model was used since the I^2^=0%. The pooled results showed that Flu + Mon significantly reduced cough recurrence

The forest plot of the total effective rate and cough recurrence rate between the Mon + Flu and Flu groups is shown in Fig. 3A-B. Because the heterogeneity among these studies was not significant (I^2^ < 50%, *P* > 0.05), based on the heterogeneity test, the fixed-effect model was used to merge the results. The pooled results showed that Flu + Mon significantly improved the total efficacy rate (RR (95% CI) = 1.30 (1.19, 1.43), *P* < 0.001) and reduced cough recurrence (RR (95% CI) = 0.51 (0.31, 0.86), *P* = 0.01).

**Fig. 4:**
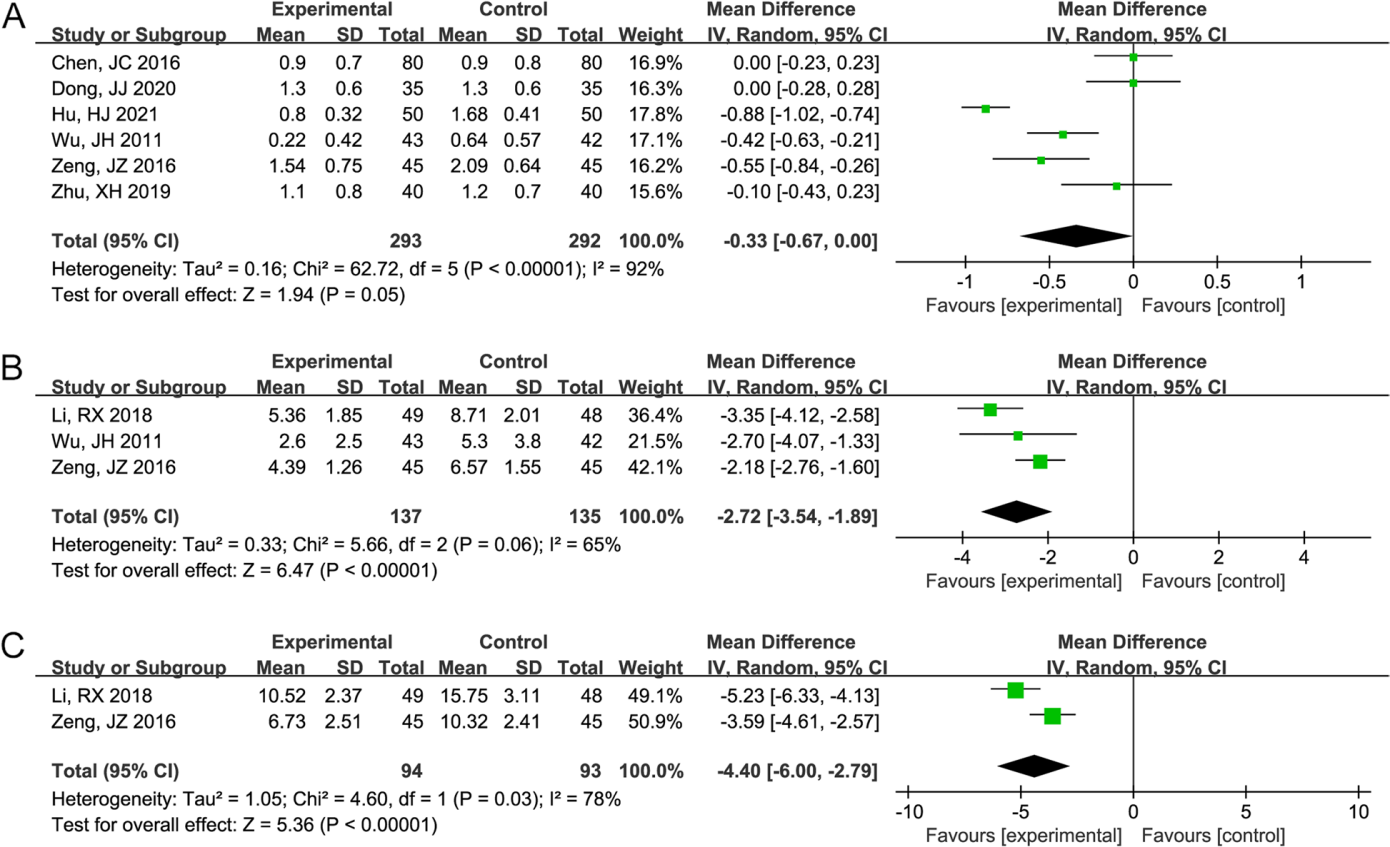
The result of the forest plot analysis for the cough symptom score, cough relief time, and cough disappearance time between the Mon + Flu group and the Flu group. **A **The result of the forest plot analysis for the cough symptom score between the Mon + Flu group and the Flu group. A random-effect model was used since the I^2^=92%. The pooled results showed that Flu + Mon significantly reduced the cough remission time. **B **The result of the forest plot analysis for cough relief time between the Mon + Flu group and the Flu group. A random-effect model was used since the I^2^=65%. The pooled results showed that Flu + Mon significantly reduced the cough relief time. **C **The result of the forest plot analysis for cough disappearance time between the Mon + Flu group and the Flu group. A random-effect model was used since the I^2^=78%. The pooled results showed that Flu + Mon significantly reduced the cough disappearance time

A forest plot of cough symptom score, cough relief time, and cough disappearance time between the Mon + Flu and Flu groups is shown in Fig. 4A-C. Because the heterogeneity among these studies was significant (I^2^ > 50%, *P* < 0.05), the random-effect model was used to merge the results. The pooled results showed that the cough symptom score (WMD (95% CI) = -0.33 (-0.67, 0.00), *P* = 0.05), the cough relief time (WMD (95% CI) = -2.72 (-3.54, -1.89) days, *P* < 0.001) and cough disappearance time (WMD (95% CI) = -4.40 (-6.00, -2.79) days, *P* < 0.001) in Mon + Flu group were significantly lower than those in the Flu group.

**Fig. 5:**
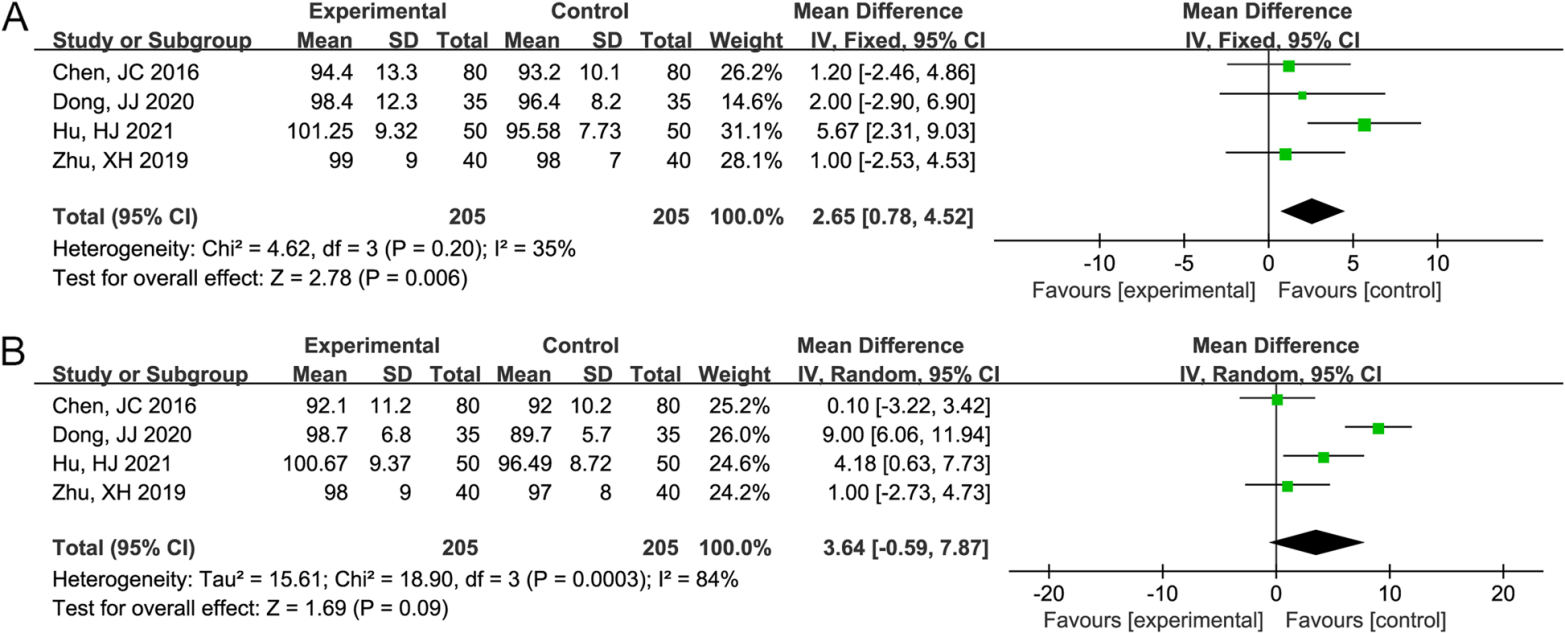
The result of the forest plot analysis for percentage of forced expiratory volume in 1 second (FEV1%) and percentage of predicted peak expiratory flow (PEF%) between the Mon + Flu group and the Flu group. **A **The result of the forest plot analysis for FEV1% between the Mon + Flu group and the Flu group. A fixed-effect model was used since the I^2^=35%. The pooled results showed that Flu + Mon significantly improved the FEV1%. **B **The result of the forest plot analysis for PEF% between the Mon + Flu group and the Flu group. A random-effect model was used since the I^2^=84%. The pooled results showed that the PEF% in Mon + Flu group was improved than that of the Flu group but without statistical significance

A forest plot of FEV1% and PEF% between the Mon + Flu and Flu groups is shown in Fig. 5A-B. Since the heterogeneity among these studies associated with FEV1% was not significant (I^2^ = 35%, *P* = 0.20), the pooled results showed that, based on the fixed-effect model, the FEV1% recovery in Mon + Flu group was significantly better than that of the Flu group (WMD (95%CI) = 2.65 (0.78, 4.52) %, *P* = 0.006). The pooled results by a random-effect (I^2^=84%) showed that, the PEF% in Mon + Flu group was improved than that of the Flu group but without statistical significance (WMD (95%CI) = 3.64 (-0.59, 7.87) %, *P* = 0.09).

**Fig. 6:**
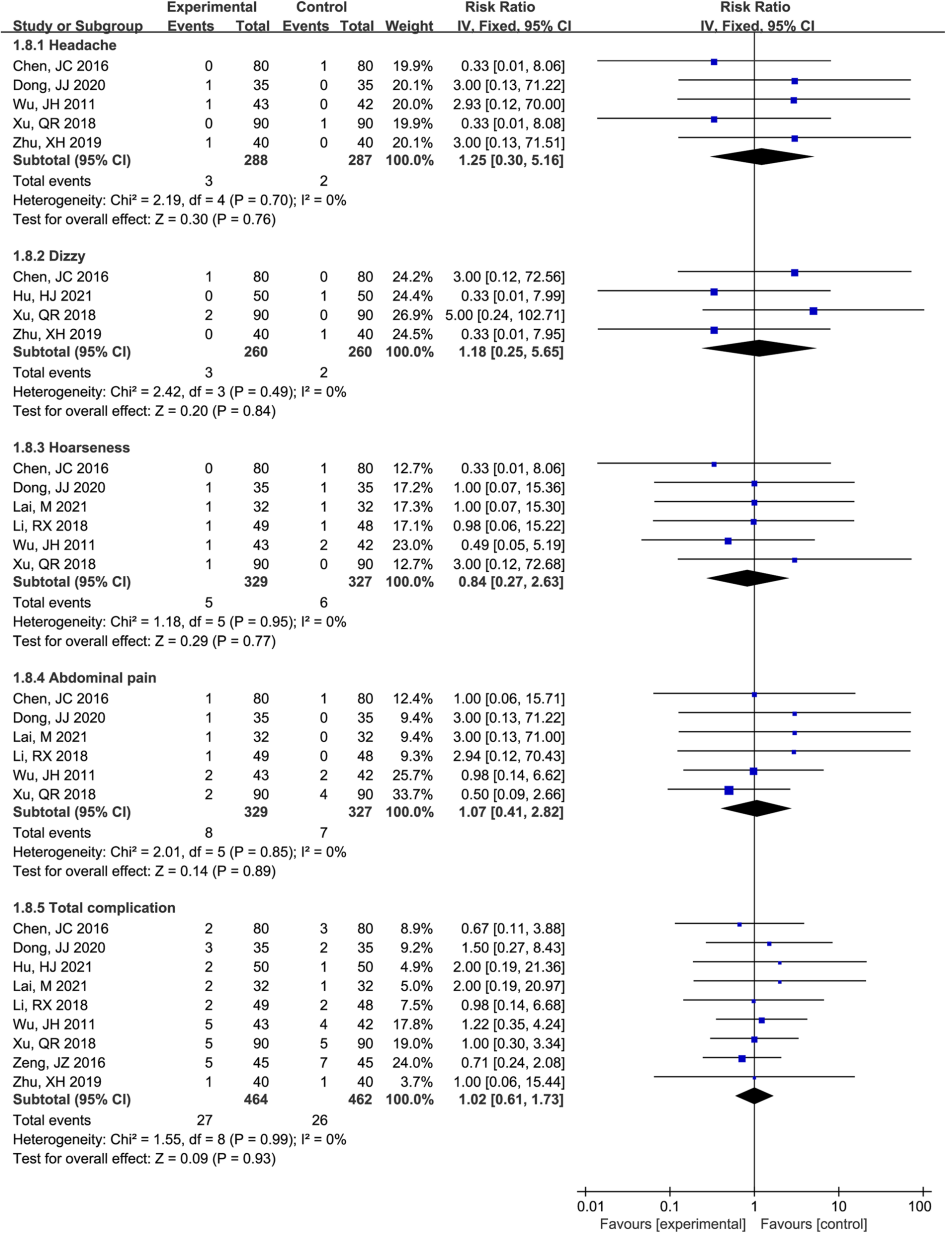
The result of the forest plot analysis for headache, dizzy, hoarseness, abdominal pain, and total complications between the Mon + Flu group and the Flu group. A fixed-effect model was used since the I^2^=0%. The pooled result indicates the incidence of headache, dizziness, hoarseness, abdominal pain and total complications were not significantly different between groups

The forest plot of headache, dizziness, hoarseness, abdominal pain, and total complications between the Mon + Flu and Flu groups showed no significant heterogeneity among these studies (I^2^ = 0%, *P* > 0.05), and the pooled result differences were not statistically significant, indicating the incidence of headache, dizziness, hoarseness, abdominal pain and total complications were not significantly different between groups (*P* > 0.05) (Fig. 6).

### Meta-regression

In order to explore the source of heterogeneity, regression analysis was used to evaluate the outcome with obvious heterogeneity. Except for cough symptom score, the number of included literatures for other indicators (cough remission time, cough disappearance time, and PEF%) was 5 or less, which was not suitable for meta regression. Therefore, meta-regression was only performed to identify the heterogeneity source on cough symptom score. The effects of sample size and treatment cycle on the heterogeneity of cough symptom score were evaluated. It was found that neither sample size (*P*=0.757) nor treatment cycle (*P*= 0.587) was significant factors influencing heterogeneity. The heterogeneity source is unclear.

### Sensitivity and publication bias analyses

Since cough appearance time was reported in only two studies, sensitivity and publication bias analyses were not available for this outcome. As shown in Table 2, the results of the sensitivity analysis suggest that the meta-analysis results of relaxation rate, cough score, FEV1%, and PEF% were unstable, and the combined results of other outcome indicators were stable. The symmetric funnel plot demonstrated no apparent publication bias (Figure S1), and Egger's tests found there was no significant publication bias in the included literature for any of the indicators (*P* > 0.05).

**Table 2:** Outcomes of the sensitivity analysis and test of publication bias

Outcomes	No. of studies	Sensitivity analysis		Egger’ s test
		HR/ORs (95% CI)	Robust	*P* value
Total effective rate	5	1.30 (1.18, 1.43) to 1.34 (1.20, 1.50)	Yes	0.087
Relapse rate	6	0.42 (0.22, 0.79) to 0.60 (0.34, 1.05)	No	0.933
The score of cough	6	-0.88 (-1.02, -0.74) to 0.00 (-0.28, 0.28)	No	0.055
Cough remission time	3	-3.19 (-3.87, -2.52) to -2.26 (-2.80, -1.72)	Yes	0.732
FEV1%	4	1.29 (-0.97, 3.54) to 3.30 (1.09, 5.50)	No	0.668
PEF%	4	1.73 (-0.73, 4.18) to 4.84 (0.13, 9.55)	No	0.242
Headache	5	1.00 (0.20, 4.90) to 1.73 (0.35, 8.46)	Yes	0.060
Dizzy	4	0.69 (0.11, 4.33) to 1.77 (0.29, 10.77)	Yes	0.346
Hoarseness	6	0.70 (0.21, 2.37) to 1.00 (0.27, 3.63)	Yes	0.474
Abdominal pain	6	0.96 (0.35, 2.67) to 1.58 (0.48, 5.19)	Yes	0.080
Total complication	9	0.98 (0.57, 1.71) to 1.15 (0.63, 2.09)	Yes	0.148

*HR* Hazard ratio, *OR* Odds ratio, *CI* Confidence interval, *FEV1%* Percentage of forced expiratory volume in 1 second, *PEF%* Percentage of predicted peak expiratory flow

## Discussion

CVA is a relatively common respiratory disease in pediatrics, which is a special type of asthma. After the onset of the disease, children can appear repeated cough symptoms, if the condition is not controlled in time, it is easy to progress into typical bronchial asthma [29]. Flu is a well-established inhaled steroid used as a preventative agent in controlling asthma symptoms [18]. A previous study showed that Flu, which suppresses eosinophilic airway inflammation, is an effective therapy for CVA, since eosinophilic airway inflammation plays an important role in CVA [30]. Chervinsky et al. showed that FEV1%, forced vital capacity, and forced expiratory flow measurements at the mid-expiratory phase at weekly visits throughout the study revealed that Flu was more efficacious than placebo in maintaining asthma control [31]. However, Kawai et al. indicated that after two weeks of Mon treatment, cough symptoms did not improve in 14 patients but disappeared two weeks after additional treatment with Flu, which further indicated the value of Mon + Flu in CVA [32]. Mon is a potent leukotriene receptor antagonist that allows dose-related asthma improvement [33]. It has been proven that both Mon and Flu can prevent recurrent wheezing CVA, but the combined effect of Mon + Flu is more effective [34]. Although Flu alone or Flu + Mon are commonly used for the clinical treatment of CVA, the efficacy and safety of Mon + Flu vs. Flu in the treatment of CVA remain controversial [12, 14, 15]. Therefore, this study compared the efficacy and safety of Mon+Flu with Flu in the treatment of CVA based on a meta-analysis, with a view to providing new insights into the clinical treatment of CVA.

One of the most important inflammatory mediators in the pathogenesis of CVA is leukotriene, which plays an important role in the airway inflammatory cascade [35]. It was found that the amount of leukotriene and its receptor in CVA patients was significantly higher than that in normal people [36]. Leukotriene can not only promote airway smooth muscle contraction, but also increase airway vascular permeability and aggravate mucosal edema [37]. Mon is a potent leukotriene receptor antagonist, which can effectively inhibit the binding of leukotriene to the receptor and block its biological effect, thereby relaxing airway smooth muscle, repairing swollen mucosa, reducing airway hyperresponsiveness, improving lung function and relieving cough [38]. In this study, we included nine articles from our online database search. The meta-analysis showed that, compared with Flu alone, Flu + Mon significantly improved the treatment efficiency, improved FEV1%, and reduced the recurrence rate, cough remission time, and cough disappearance time in CVA. Moreover, the risk of adverse reactions did not significantly increase. Thus, we speculated that Flu + Mon was more effective and safer for CVA treatment than Flu alone.

There were some advantages to our study analysis: i) all enrolled studies were RCTs with low methodological heterogeneity and medium risk level of bias; ii) the differences in research design, clinical information, and statistical heterogeneity of outcomes among the included studies were small; and iii) the results were highly reliable, because there was no significant publication bias among the studies.

However, the current study had some limitations. Specifically, i) small sample size; ii) no quantitative methods such as meta-regression or subgroup analysis were used to identify the source of heterogeneity; and iii) the enrolled studies were all from China, and the extrapolation of meta-analysis results was affected.

## Conclusions

In conclusion, by integrating 9 studies, we obtained a more comprehensive and objective results that Mon + Flu is relatively effective and safe regimen for treating CVA in children. This study may provide novel insights into the clinical treatment of CVA. Further prospective cohort studies with high quality and large samples are needed to verify the authenticity of the results.

### Supplementary Information

**Additional file 1: Figure S1.** Funnel plot. A, the total effective rate. B, cough recurrence rate. C, the cough symptom score. D, cough relief time. E, cough disappearance time. F, percentage of forced expiratory volume in 1 second (FEV1%). G, percentage of predicted peak expiratory flow (PEF%). H, headache. I, dizzy. J, hoarseness. K, abdominal pain. L, total complications.

## Data Availability

All data generated or analysed during this study are included in this published article (and its supplementary information files).
